# Admission Serum Inflammatory and Injury Biomarkers Are Associated with In-Hospital Mortality in Neurological Inpatients with Confirmed SARS-CoV-2 Infection: The Brain-COVID Cohort Study

**DOI:** 10.3390/cimb48020228

**Published:** 2026-02-20

**Authors:** Justyna Zielińska-Turek, Wojciech Czyżewski, Grzegorz Turek, Tomasz Lyson, Jan Gajewski, Patrycja Gierszon, Michał Turek, Klaudia Kuś-Budzyńska, Małgorzata Dorobek

**Affiliations:** 1 Department of Neurology, National Medical Institute of the Ministry of Interior and Administration, 02-507 Warsaw, Poland; jzturek@gmail.com (J.Z.-T.); malgorzata.dorobek@pimmswia.gov.pl (M.D.); 2 Department of Neurosurgery, Maria Sklodowska-Curie National Research Institute of Oncology, ul. W.K. Roentgena 5, 02-781 Warsaw, Poland; 3 Department of Didactics and Medical Simulation, Medical University of Lublin, Chodźki 4, 20-093 Lublin, Poland; 4 Department of Neurosurgery, Postgraduate Medical Centre, Brodnowski Masovian Hospital, 03-242 Warsaw, Poland; turek.grz@gmail.com (G.T.); michal.turek1997@gmail.com (M.T.); 5 Department of Neurosurgery, Medical University of Bialystok, M. Sklodowskiej-Curie 24A, 15-276 Bialystok, Poland; lyson_t@vp.pl; 6 Department of Human Biology, Józef Piłsudski University of Physical Education, 00-968 Warsaw, Poland; jan.gajewski@awf.edu.pl; 7 Faculty of Medical Sciences, University of Social and Medical Sciences in Lublin, 20-102 Lublin, Poland; pati6777@wp.pl; 8 Department of Neurosurgery, Medical University of Lublin, 20-954 Lublin, Poland; kkusbudzynska@gmail.com

**Keywords:** COVID-19, neurological disorders, systemic inflammation, interleukin-6, ferritin, LDH, troponin I, biomarkers

## Abstract

Patients hospitalized with neurological disorders may be at increased risk of adverse outcomes when infected with SARS-CoV-2. We evaluated whether early routine serum inflammatory and injury markers obtained at hospital admission are associated with in-hospital mortality in this subgroup. This single-center observational cohort included 460 consecutive adult inpatients admitted for neurological disorders with SARS-CoV-2 infection confirmed on admission or during hospitalization. Serum IL-6, LDH, ferritin, hs-troponin I, CRP, procalcitonin, and D-dimers measured within 6 h of hospital admission for neurological disorder were analyzed and compared between survivors and non-survivors. Non-survivors had higher IL-6, LDH, ferritin, and hs-troponin I (all *p* < 0.001). In multivariable analysis, LDH, ferritin, IL-6, and hs-troponin I were independently associated with mortality. We conclude that in neurological inpatients with confirmed SARS-CoV-2 infection, elevated early IL-6, LDH, ferritin, and hs-troponin I are associated with in-hospital mortality. These markers likely reflect systemic disease severity rather than CNS-specific neuroinflammation and may support early risk stratification in this population.

## 1. Introduction

COVID-19, caused by the beta-coronavirus SARS-CoV-2, is a systemic disease with a wide clinical spectrum—from asymptomatic infection to severe inflammatory and coagulopathic syndromes involving the lungs, cardiovascular system, and central nervous system (CNS) [1,2]. Although respiratory symptoms dominate, neurological involvement has been increasingly recognized as both a target and a modulator of immune dysregulation [2,3,4,5].

A growing body of evidence indicates that an uncontrolled immune response—known as a “cytokine storm”—plays a key role in disease deterioration [6,7,8]. Overproduction of pro-inflammatory cytokines, particularly interleukin-6 (IL-6), induces acute-phase reactants such as C-reactive protein (CRP), procalcitonin (PCT), ferritin, and lactate dehydrogenase (LDH), contributing to multi-organ dysfunction and poor prognosis [9,10,11]. IL-6 may influence multiple organ systems, and neurological complications have been reported in severe COVID-19; however, routine serum markers primarily reflect systemic disease severity, which can lead to encephalopathy, delirium, or stroke-like syndromes [7,12,13].

Patients with pre-existing neurological disorders may be particularly vulnerable to severe COVID-19. Chronic neurological diseases are often accompanied by autonomic dysregulation, impaired immune responses, and vascular comorbidities that may amplify systemic inflammation [2,14,15,16]. Identifying early routine biomarkers of systemic inflammation and tissue injury (IL-6, LDH, ferritin, CRP, D-dimers, hsTnI) may help recognize neurological inpatients at higher risk of poor outcomes [17,18,19,20,21].

COVID-19 triggers systemic inflammatory and coagulation responses that are reflected in routinely measured serum biomarkers (e.g., IL-6, LDH, ferritin, CRP, and D-dimers). We therefore assessed whether early admission biomarker levels are associated with in-hospital mortality among neurological inpatients with confirmed SARS-CoV-2 infection [22,23].

Previous studies have shown that elevated IL-6, ferritin, LDH, and hsTnI levels are associated with severe or fatal COVID-19 in general populations [16,24,25,26,27]; however, the prognostic value of these markers in patients with neurological comorbidities remains unclear.

Therefore, we evaluated whether routinely available admission serum biomarkers are associated with in-hospital mortality in neurological inpatients with confirmed SARS-CoV-2 infection.

## 2. Materials and Methods

From 1 March 2020 to 31 December 2024, we consecutively enrolled 460 adult patients (≥18 years; 235 women, mean age 71.1 ± 17.4 years; 225 men, mean age 66.8 ± 15.2 years) admitted for neurological disorders with confirmed SARS-CoV-2 infection. Thirty patients had ≥2 neurological diagnoses. This investigation, hereafter referred to as the BRAIN-COVID Study (Biomarkers of Risk and Inflammation in Neurological COVID), aimed to explore the relationship between systemic inflammatory responses and outcomes in patients with coexisting neurological disorders and COVID-19 infection. Baseline clinical assessment and blood sampling were performed within 6 h of hospital admission for a neurological disorder. SARS-CoV-2 infection was confirmed either on admission or during hospitalization; all analyses refer to biomarkers obtained within the first 6 h of hospital admission. Neurological diagnoses were made by board-certified neurologists using magnetic resonance, computed tomography and Doppler ultrasonography (otherwise, chest X-ray was done). Patients testing positive for influenza A/B or Respiratory Syncytial Virus, those with procalcitonin >0.5 ng/mL plus bacterial pneumonia on computed tomography, and individuals on long-term antibiotics or immunosuppressants were excluded.

**Figure 1:**
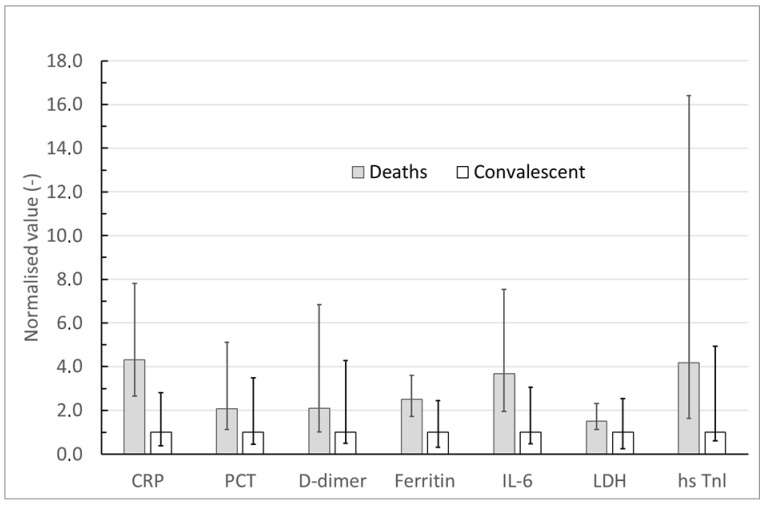
Concentrations of inflammatory proteins in two groups of 460 COVID-19 patients: non-survivors and survivors.

On admission, respiratory status and consciousness were scored with MEWS (Modified Early Warning Score), GCS (Glasgow coma scale) and mRS (Modified Rankin Scale). Within six hours, blood was drawn for CBC and plasma CRP, PCT, LDH, ferritin, IL-6 (interleukin-6), D-dimers (VIDAS D-dimer, normal ≤500 µg/L) and hsTnI (Abbott hsTnI). All biochemical parameters were analyzed in the certified hospital laboratory. Reference (normal) ranges were laboratory- and assay-specific, provided by the manufacturers of the diagnostic kits, and all units were standardized across the manuscript. We calculated median biomarker values (and ranges) for survivors versus non-survivors (Table 1, Figure 1) and across individual neurological diagnoses (Table 2).

### Statistical Analysis

Variable distributions were right-skewed (Kolmogorov–Smirnov), so we reported medians and interquartile ranges. Between-group differences were assessed with Mann–Whitney U (Glass rank-biserial effect size) and chi-square tests (Cramer’s φ), applying Bonferroni-corrected *p*-values. Backward stepwise logistic regression (Table 4) evaluated mortality risk based on age and admission serum biomarkers (log-transformed). There were no missing data for the biomarker variables included in the primary model.

## 3. Results

Neurological status was assessed on admission using MEWS, GCS and mRS. Due to the ordinal and bounded nature of these scales, results are summarized descriptively and were not used as primary baseline predictors in the prognostic model. Out of 460 patients, the most common and concomitant neurological diagnosis was ischemic stroke, affecting 240 patients (52.2%): 123 men (mean age 68.6 ± 13.7 years) and 117 women (mean age 73.2 ± 14.8 years). Twenty patients out of that group underwent thrombolysis and 15 received mechanical thrombectomy. Other diagnoses included dementia (6.5%), epilepsy (6.5%), central nervous system tumors (4.5%), hemorrhagic stroke (3.9%), and miscellaneous conditions (20.8%) (Table 2). Thirty patients (6.1%) had overlapping neurological disorders (Table 3). The most frequent non-neurological comorbidity was arterial hypertension (251 patients), followed by diabetes mellitus (112), atrial fibrillation (83), and malignancy (47). Less common were renal failure (40), nicotine addiction (30), asthma (19), significant obesity (18), and alcohol addiction (15).

Thrombosis and embolism were found to be main factors in 43 out of the 240 patients with ischemic stroke diagnosed with angio-computed tomography imaging. A cardiogenic mechanism of stroke was suspected in 61 out of the 240 patients (those presenting with arrhythmias or atrial fibrillation). Forty-nine patients presented with coexisting hypertension, dyslipidaemias, type 2 diabetes, or an addiction to nicotine. The remaining patients with ischemic stroke presented with end-stage renal failure and/or disseminated neoplastic disease. Dementia was the second most frequent neurological diagnosis. Patients with dementia were significantly older than the overall cohort (mean age 82.1 ± 7.7 years, range 67–99 vs. 71 ± 16.8 years, range 19–99; *p* < 0.05) and carried multiple comorbidities likely to worsen their prognosis, as expected.

The most common symptom was fever (28%), followed by dyspnea (23%), cough (15%), myalgia and headache (each 9%), vomiting (7%), dizziness (6%), changes in taste or smell (5%), diarrhea (4%), and constipation (2%). Chest computed tomography scans revealed interstitial densities, crazy-paving patterns, ground-glass opacities, and exudates; these findings are typical of advanced COVID-19. However, a substantial number of patients presented with normal chest computed tomographs despite severe clinical illness.

Management of advanced COVID-19 prioritized respiratory support. Among 460 patients, 75 received high-flow oxygen therapy and 29 required mechanical ventilation. Despite intensive-care treatment, mechanically ventilated patients faced a 90% mortality rate. Overall, 115 of the 460 patients (25%) died. Mortality among the 30 patients with multiple neurological conditions did not differ significantly from the general cohort (log-linear analysis), nor did their inflammatory-protein concentrations (Mann–Whitney U test).

In the initial phase of the pandemic, chloroquine played a major role in treatment. In our study group, it was administered to 57 patients. Azithromycin (35%) and/or ceftriaxone (41%) were added for those with interstitial pneumonia. Nine chloroquine-resistant patients received lopinavir/ritonavir; seven of these nine died, and three exhibited laboratory evidence of pancreatitis. Following emerging evidence for remdesivir, we revised our protocol to administer remdesivir—either alone or with convalescent plasma—during the first seven days of symptoms, then switch to tocilizumab for the following week. In the cohort of 40 patients treated with remdesivir, there were only four deaths. Notably, survivors in this group were discharged 3.3 days sooner on average than survivors who did not receive the antiviral. Outcomes in the 20 patients given convalescent plasma were similar: four deaths, and survivors left the hospital 4.1 days earlier than their untreated counterparts from the pandemic’s initial phase. Overall, 40 patients received remdesivir, 20 received convalescent plasma, and two received tocilizumab. Additionally, 36% of patients on oxygen therapy were supplemented with dexamethasone. Interventions such as mechanical ventilation, dexamethasone and lopinavir/ritonavir were used predominantly in patients with severe clinical deterioration and markedly elevated inflammatory markers. Therefore, any observed associations between these interventions and mortality should be interpreted as reflecting disease severity and treatment indication rather than baseline prognostic factors. Therefore, these variables reflect disease severity and treatment indication rather than independent causal effects on mortality.

For the entire cohort, median levels of C-reactive protein, ferritin, D-dimers, and lactate dehydrogenase exceeded normal reference ranges, whereas median values for IL-6, lymphocyte count, procalcitonin, and high-sensitivity troponin I remained within normal limits (Table 1). In the three most common neurological conditions accompanying COVID-19, inflammatory mediator levels varied markedly (Table 2). In ischemic stroke, D-dimer concentrations exceeded 1726 µg/L—over three times the upper reference limit (≤500 µg/L)—while procalcitonin remained within normal range and both C-reactive protein and ferritin rose threefold. In dementia, procalcitonin again stayed normal, whereas C-reactive protein increased sixfold and ferritin fourfold. Among epilepsy patients, C-reactive protein was elevated fourfold, and interleukin-6 and high-sensitivity troponin I were also significantly raised.

The concentrations of all the tested inflammatory proteins turned out to be significantly higher in patients who required mechanical ventilation (*p* < 0.05), and in those who were treated with dexamethasone (*p* < 0.001) or lopinavir and ritonavir (*p* < 0.05), compared to those who did not require such vigorous therapy (Mann–Whitney test). Ferritin, IL-6, LDH, and hs-troponin I showed the largest between-group differences (η^2^ > 0.14; Table 4, Figure 1), with survivors having significantly lower plasma levels than non-survivors.

Median hsTnI was substantially higher in non-survivors and rose with patient age. In multivariable analysis combining age with log-transformed biomarkers, LDH showed the highest odds ratio, followed by ferritin, hsTnI, and IL-6 (Table 4).

**Figure 2:**
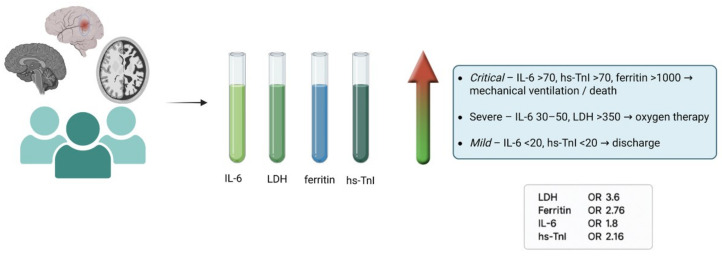
Predictive Role of Inflammatory Biomarkers in Neurological COVID-19: Clinical Outcome Model. Created in BioRender. G, P. (2026) BioRender.com/63sryx0.

The most accurate model combined age with the logarithms of key inflammatory markers (Table 4). As summarized in Figure 2, LDH showed the highest odds ratio (3.6), followed by ferritin (2.76), hs-troponin I (2.16), and IL-6 (1.8).

Non-survivors were significantly older than survivors (75.8 ± 11.4 vs. 65.3 ± 16.9 years; Z = 6.7, *p* < 0.0001; RG = 0.45). Gender had no meaningful impact on inflammatory-marker levels in either group (all RG < 0.1). Survival did not differ by sex (women: 79% survived; men: 73% survived; a nonsignificant difference (χ^2^ = 1.48, *p* = 0.22; φ = 0.061)). We did not observe a significant association between sex and either inflammatory marker levels or in-hospital mortality.

Across all markers, median concentrations in survivors were below the overall cohort medians, whereas non-survivors’ medians exceeded them. Within the survivor subgroup, only procalcitonin (0.07 ng/mL) and hs-troponin I (7.5 pg/mL) reached the upper normal limits; all other parameters remained only slightly elevated (Table 1).

## 4. Discussion

The BRAIN-COVID Study shows that early routine serum inflammatory and injury markers are associated with in-hospital mortality in neurological inpatients with confirmed SARS-CoV-2 infection.

While similar markers have been linked to outcomes in general COVID-19 populations, our findings extend these observations to neurological inpatients, a clinically vulnerable group frequently burdened by advanced age and comorbidities. These associations likely reflect systemic disease severity in a clinically vulnerable neurological inpatient population. We do not exclude that some neurological conditions may independently contribute to systemic inflammation; however, our data do not allow disentangling these effects from COVID-19-related systemic responses [28].

Severe SARS-CoV-2 infection is associated with systemic immune dysregulation and increased inflammatory markers, which have been linked to adverse outcomes in general COVID-19 populations [29].

Our analysis included IL-6, which is overproduced to recruit immune cells against the virus [7]. Abnormal leukocyte recruitment affects multiple organs—particularly the lungs—leading to acute respiratory distress syndrome [12,13,14,15]. Zhou et al. showed that, in addition to IL-6, elevated D-dimer, ferritin, and lactate dehydrogenase levels may contribute to the cytokine storm and increase the risk of death [16]. Zeng et al. confirmed that higher IL-6 levels correlate with increased mortality in COVID-19 patients [13]. Excessive IL-6 secretion may also lead to lymphopenia. Another adverse effect is the abnormal activation of the coagulation cascade, which can precipitate sudden clinical deterioration [17,18,19,20,21].

Considering the pivotal role of excess IL-6 in driving harmful biological reactions, this cytokine can be used to monitor COVID-19 patients and predict clinical deterioration. Several IL-6 cutoffs have been proposed as diagnostic thresholds for identifying increased risk of deterioration or death. Elshazli et al. and Guirao et al. suggested thresholds of 22.9 pg/mL and 34.9 pg/mL, respectively, to predict the need for Intensive Care Unit transfer [24,25]. Riveiro-Barciela et al. reported that an IL-6 concentration of 64 pg/mL predicted the requirement for high-flow oxygen therapy (hazard ratio 18) [26]. According to Mandel et al., an IL-6 level of 163.4 pg/mL strongly predicted death, with 91.7% sensitivity and 57.6% specificity [27]. In our study, IL-6 also emerged as a robust—but not the strongest—predictor of death. Using backward stepwise logistic regression, the odds ratio for IL-6 was 1.8. The median IL-6 concentration was significantly higher in non-survivors than in survivors (39.75 pg/mL vs. 10.1 pg/mL).

High-sensitivity troponin I was measured in all patients, irrespective of acute coronary syndrome symptoms, because COVID-19-related chest pain can mimic heart failure. Elevated hsTnI indicates cellular damage and may occur in viral and bacterial infections, sepsis, central nervous system vascular diseases, acute respiratory distress syndrome, and renal insufficiency. Zhou et al. reported that hsTnI concentrations rose in advanced or progressive COVID-19 cases, especially in patients who died. In our cohort, hsTnI was significantly higher in non-survivors and remained independently associated with mortality in the multivariable model (Table 3).

Lippi et al. demonstrated that procalcitonin assessment may help determine the risk of COVID-19 complications, clinical deterioration, and death [14]. In our study, survivors had procalcitonin levels at the upper limit of the normal reference range (0.08 ng/mL), whereas non-survivors exhibited values twice as high. Contrary to Lippi et al., our backward stepwise logistic regression showed that procalcitonin was not a reliable predictor of death (*p* > 0.05). Rodelo et al. performed a retrospective analysis of patients with suspected infection or sepsis and found that non-survivors had a median D-dimer level of 2489 ng/mL, corresponding to an odds ratio for death of 3.03 (95% CI, 1.38–6.62) [30]. In our cohort, D-dimer concentrations were also elevated in non-survivors, but we observed no statistically significant relationship with mortality in the regression analysis (*p* > 0.05).

Lactate dehydrogenase is an enzyme that catalyzes the conversion of lactate to pyruvate and is therefore present in nearly all cells. Blood LDH measurements are routinely used to monitor tissue damage in various disorders, including liver disease, interstitial lung disease, and SARS-CoV-2-induced illness. LDH is also a key biomarker of activity and severity in idiopathic pulmonary fibrosis. In our study, non-survivors exhibited a dramatic increase in LDH concentration, reflecting extensive lung injury and serving as an important prognostic marker. Bartziokas et al. similarly reported that LDH levels rise in critically ill COVID-19 patients as disease severity and lung damage progress, and that elevated plasma LDH is closely linked to COVID-19 mortality [31]. Furthermore, in our cohort, elevated LDH was the strongest predictor of death, with an odds ratio of 3.6.

Ferritin is another protein that influences COVID-19 severity. Zhou et al. found that patients with advanced COVID-19 had elevated serum ferritin levels, with the “very severe” form showing medians of 1006.2 ng/mL (IQR, 408.3–1988.3) versus 291.1 ng/mL (IQR, 102.1–648.4) in “severe” cases [32]. Chen et al. reported that patients who died from COVID-19 exhibited high ferritin levels from admission through their hospital stay, with median concentrations exceeding the assay’s upper detection limit after day 16 [33]. Our findings align with these observations: non-survivors had significantly higher serum ferritin levels than survivors. Backward stepwise logistic regression identified elevated ferritin as a strong predictor of death (odds ratio, 2.76).

**Figure 3:**
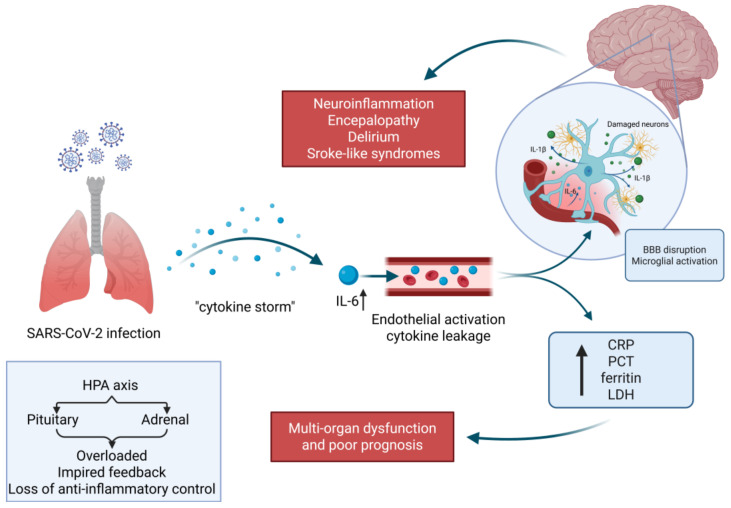
SARS-CoV-2 infection may trigger systemic inflammation and tissue injury reflected by elevated IL-6, CRP, ferritin, and LDH, which are associated with worse in-hospital outcomes. Created in BioRender. Turek, M. (2026) BioRender.com/hfrs595.

Our study did not include CNS-compartment biomarkers (e.g., CSF markers) or longitudinal neurological endpoints. Therefore, the observed associations should be interpreted as reflecting systemic inflammation and tissue injury rather than CNS-specific neuroinflammation. Future studies incorporating neurological follow-up and CNS biomarkers are needed to address mechanistic hypotheses (Figure 3).

Advanced age is well-established predictor of COVID-19 severity. Nevertheless, our findings demonstrated only a modest impact of age on mortality risk, with an odds ratio barely exceeding 1. This unexpected result likely reflects the substantial burden of preexisting conditions—such as diabetes mellitus, cardiovascular disease, chronic kidney disease, hypertension, obesity, and lifestyle factors—in our study population. These comorbidities may have independently increased mortality risk regardless of age [34,35,36,37]. In summary, COVID-19 infection in patients with preexisting or coexisting comorbidities carries a high risk of mortality, multi-organ dysfunction with respiratory failure, encephalopathy, acute cardiac injury, renal failure, and damage to other organs [32]. Guo et al. developed the MuLBSTA score (multilobular infiltrates, lymphocytes ≤0.8 × 10^9^/L, bacterial infection, smoking, hypertension, and age ≥60 years) to help predict outcomes in COVID-19 patients [35].

Interestingly, the risk of death varied among patient subgroups with ischemic stroke, epilepsy, dementia, or other neurological conditions. Dementia patients had highest mortality, proximately 52%—but this group also had the highest median age (84.1 years) and a heavy burden of comorbidities. In contrast, the epilepsy subgroup had a relatively low mortality risk. These patients were younger (median age 62.1 years) and experienced few deaths, most of which were related to refractory status epilepticus.

In our cohort of 460 patients, treatment varied according to clinical condition. Seventy-five patients received high-flow oxygen therapy, and 29 required mechanical ventilation. Mortality in the ventilated group was 90%. Mortality was high among mechanically ventilated patients; however, ventilation is a marker of severe disease course and cannot be interpreted as an independent baseline predictor. Importantly, these therapies were reserved for patients with the most severe disease and markedly elevated inflammatory markers, indicating that the observed associations likely reflect confounding by indication rather than independent causal effects. Notably, all tested inflammatory proteins were significantly higher (*p* < 0.05) in patients requiring ventilation than in those who did not. This finding highlights the interrelationship between cytokine elevation at admission and the subsequent need for artificial ventilation, reinforcing the prognostic value of cytokine measurement in neurological patients with COVID-19. Pro-inflammatory cytokine determination may also play a key role in predicting the need for mechanical ventilation during hospitalization. In a study of 1042 patients hospitalized with COVID-19, Nicholson et al. found that mortality among mechanically ventilated patients increased with age: near 0% in those under 35 years and 75% in those 75 years or older [38]. In contrast, we found no significant relationship between age and mortality risk among ventilated patients, which may be related to the small size of the group (29 patients) in which mechanical ventilation was required.

At the start of the pandemic, our institution frequently used chloroquine in more than half of treated patients. After publications questioned its efficacy, chloroquine was withdrawn in favor of other agents [39]. Surprisingly, chloroquine therapy did not affect mortality risk in our multivariable logistic regression analysis. Similarly, azithromycin and/or ceftriaxone—used as adjunctive therapy in interstitial pneumonia—had no impact on mortality. In contrast, treatment with lopinavir and ritonavir increased the odds of death to 15.4. These drugs were reserved for patients resistant to chloroquine and, notably, that subgroup had significantly higher admission cytokine concentrations than the rest of our cohort. These findings may be limited to the small sample size. Meini et al. reviewed lopinavir and ritonavir in COVID-19 and concluded that evidence is scarce and low-quality, with guidelines ranging from recommendations against their use to cautious support. They argued that these drugs should not be abandoned despite mixed trial results [40]. With emerging evidence on remdesivir, our protocol shifted to remdesivir—alone or with convalescent plasma—followed by tocilizumab. In our study, remdesivir did not increase mortality risk, and Beigel et al. demonstrated that remdesivir shortened recovery time compared to placebo in hospitalized COVID-19 patients [41]. We supplemented oxygen therapy with dexamethasone in 36% of patients, and this group had an odds ratio of death of 2.1 compared to those who did not require corticosteroids. Similar to the ritonavir-treated subgroup, patients receiving dexamethasone had significantly higher admission cytokine levels. Ahmed et al. cautioned against liberal corticosteroid use, noting that high doses can cause harm [42]. Conversely, the RECOVERY Collaborative Group showed that dexamethasone reduced 28-day mortality in 2104 hospitalized COVID-19 patients receiving invasive ventilation or oxygen therapy [43]. This discrepancy highlights the need for further clarification of corticosteroids’ role in COVID-19 therapy.

## 5. Limitation of the Study

Although our cohort was relatively large, only 30 patients had more than one overlapping neurological disease. Consequently, conclusions about this subgroup—particularly regarding morbidity—should be interpreted cautiously and verified in future studies with a larger sample of COVID-19 patients who have multiple neurological comorbidities. Another limitation is the high prevalence of non-neurological comorbidities—such as atrial fibrillation, renal failure, and extracranial neoplasms—in our study population. These conditions may have influenced the expression of the inflammatory proteins we measured. However, such a comorbid profile is typical in real-world COVID-19 cohorts and recruiting the patients entirely free of concomitant diseases would generate a cohort which is not representative. We measured inflammatory proteins only upon admission to assess their prognostic value. Therefore, it is difficult to determine whether their elevated concentrations directly increased mortality risk or did so indirectly through specific treatments (for example, mechanical ventilation, antiviral therapy, or corticosteroids). Additionally, we could not analyze how these inflammatory markers changed over the course of hospitalization, limiting insights into their dynamic relationship with disease progression.

## 6. Conclusions

This study demonstrated a strong association between inflammatory biomarker concentrations and COVID-19 severity in patients with pre-existing neurological disorders. Elevated IL-6, LDH, ferritin, and high-sensitivity troponin I levels at hospital admission were independently linked to an increased risk of death, highlighting their prognostic value in this vulnerable population.

Patients who required mechanical ventilation or received lopinavir/ritonavir or dexamethasone had higher inflammatory marker levels; these patterns likely reflect confounding by indication, as such interventions were reserved for the most severely ill patients.

These findings suggest that systemic inflammation and tissue injury, reflected by routinely available admission biomarkers, are associated with worse in-hospital outcomes and may support early risk stratification in this population. Monitoring IL-6, LDH, ferritin, and hsTnI may help identify high-risk patients early during hospital admission.

## Figures and Tables

**Table 1:** Outcome and concentration levels of inflammatory proteins in 460 COVID-19 patients with coexisting neurological diseases.

	**Total**	**Convalescents**	**Deaths**	**Mann–Whitney U Test**			
	**Median**	**Median**	**Median**	**U**	**Z**	***p*** **-Value**	**R_G_**
	(lower Q–upper Q)	(lower Q–upper Q)	(lower Q–upper Q)				
No.	460	325	115				
Age (years)	71 (19–99)	65.3 (19–94)	75.81 years (39–99)		6.73	<0.0001 *	0.457
C-reactive protein (reference range, local laboratory: <10 mg/L)	26.7 (5.05–73.8)	15.8 (2.7–56.9)	65.2 (39.8–118.1)	7033	−7.52	<0.0001 *	0.5115
Procalcitonin (reference range, local laboratory: <0.1 ng/mL)	0.09 (0.05–0.19)	0.07 (0.04–0.15)	0.168 (0.09–0.41)	7577	−6.86	<0.0001 *	0.4679
D-dimers (reference range, local laboratory: ≤500 µg/L)	1246 (677–2554)	1073 (526–2189)	2240 (1095–7318)	7981	−6.06	<0.0001 *	0.4193
Ferritin (reference range, local laboratory: 24–336 µg/L)	588 (301–1157)	441 (268–871)	1159 (774–1638)	5541	−7.94	<0.0001 *	0.5640
IL-6 (reference range, local laboratory: <18 pg/mL)	16.3 (5.3–39.1)	10.1 (3.85–29.5)	39.75 (20.55–77.25)	5385	−7.94	<0.0001 *	0.5672
LDH (reference range, local laboratory: 140–280 U/L)	291 (205–391)	262 (197–340.5)	397 (297–609)	6108	−7.56	< 0.0001 *	0.5320
Highly sensitive troponin I (reference range, local laboratory: <15.6 pg/mL) Female: <15 pg/mL Male: <20 pg/mL	12.2 (4.2–29.6)	7.5 (3.1–19.4)	43.6 (15.6–186.9)	5502	−8.99	<0.0001 *	0.6136
White blood cells (reference range, local laboratory <10 tys./mL)	7.43 (2.39–120.3)	7.15 (2.45–119.02)	9.41 (4.07–28.05)	4967	-	<0.0001 *	-

* *p* < 0.001 shows statistical significance.

**Table 2:** Concentration levels of inflammatory proteins and number of deaths according to different diagnoses.

	**Median (Range)**					
	Ischemic stroke	Epilepsy	Dementia	Central nervous system tumors	Hemorrhagic stroke	Others
No.	240	30	30	21	18	121
Age	73.5 years (38–98)	61.1 years (38–92)	84.1 years (67–99)	71 years (44–84)	65 years (23–84)	64.4 years (19–91)
Death (n)	40	6	18	5	10	36
C-reactive protein (reference range, local laboratory: <10 mg/L)	28.1 (0.3–347.7)	44.3 (1.6–196.1)	44.2 (2.1–184.4)	2 (0.2–177.2)	17 (0.8–191.4)	23.5 (0.2–404.6)
D-dimers (reference range, local laboratory: ≤500 µg/L)	1726 (215–94,437)	1336 (221–23,171)	1129.5 (674–22,580)	1497.5 (149–8117)	1626 (315–26,426)	1089 (181–28,384)
IL-6 (reference range, local laboratory: <18 pg/mL)	26.6 (2.03–3144)	44.8 (1.5–3430)	42.6 (2.49–243)	4.94 (1.5–144)	7.25 (0.9–81.3)	10.2 (0.5–6493)
Procalcitonin (reference range, local laboratory: <0.1 ng/mL)	0.07 (0.008–226)	0.15 (0.02–39.7)	0.15 (0.07–2.85)	0.07 (0.02–5.09)	0.08 (0.04–0.37)	0.1 (0.02–219)
Ferritin (reference range, local laboratory: 24–336 µg/L)	511 (41–3721)	951 (39–1403)	795.5 (137–2473)	560 (180–1650)	390.5 (179–2159)	560 (71–5515)
LDH (reference range, local laboratory: 140–280 U/L)	306 (71–1029)	345 (139–1180)	285.5 (180–728)	234 (187–338)	257 (120–708)	286 (112–1191)
Highly sensitive troponin I (reference range, local laboratory: <15.6 pg/mL)	15.7 (0.3–28,287)	22.65 (1.1–16,148)	19.1 (4.5–380.5)	5.9 (1.1–45.1)	11.15 (2.2–30.9)	6.3 (0–275.9)
White blood cells (reference range, local laboratory: <10^3^/mL	7.75 (2.88–121.3)	8.34 (3.5–14.68)	7.53 (2.93–28.06)	10.33 (4.7–19.85)	7.58 (4.08–18.1)	6.68 (2.87–28.41)

**Table 3:** Diagnoses in a sub-group of 30 patients presenting with more than one neurological disease concomitant with COVID-19.

	**Dementia**	**Epilepsy**	**Parkinson** **Disease**	**Multiple Sclerosis**	**Polyneuropathy**	**Myasthenia**	**SAH**	**Migraines**	**Central Nervous System** **Tumor**	**Other**
Dementia	x	1	4	x	1	0	0	x	x	1
Epilepsy	0	x	0	1	0	0	0	1	4	1
Parkinson Dis.	0	0	x	0	1	0	0	x	0	1
M. Sclerosis	0	0	0	x	0	0	0	0	0	0
Polyneuropathy	0	0	0	0	x	0	0	0	0	0
Myasthenia	0	0	0	0	0	X	0	0	0	0
SAH	0	0	0	0	0	0	x	0	0	0
Migraines	0	0	0	0	0	0	0	x	0	0
Central nervous system tumor	0	0	0	0	0	0	0	0	x	0
Ischemic Stroke	7	5	3	1	0	0	0	0	0	0

**Table 4:** Multivariable logistic regression model for in-hospital mortality based on age and admission serum biomarkers (n = 460).

	Odds Ratio	Confidence Interval (CI)	*p*
Age	1.044	1.02–1.07	0.0007 *
lnIL-6	1.8	1.28–2.20	0.0004 *
lnLDH	3.6	1.50–9.27	0.0004 *
lnTnI	2.16	1.44–2.54	<0.0001 *
lnFerritin	2.76	1.61–4.71	0.0001 *

* Logistic regression: *p* < 0.01 shows statistical significance.

## Data Availability

The original contributions presented in this study are included in the article. Further inquiries can be directed to the corresponding author.

## Abbreviations

The following abbreviations are used in this manuscript:

IL-6	interleukin-6
CRP	C-reactive protein
PCT	procalcitonin
LDH	lactate dehydrogenase
hsTnI	high-sensitivity troponin I
MEWS	Modified Early Warning Score
GCS	Glasgow coma scale
mRS	Modified Rankin Scale
